# Multiplicity of infection and genetic diversity of *Plasmodium falciparum* isolates from patients with uncomplicated and severe malaria in Gezira State, Sudan

**DOI:** 10.1186/s13071-016-1641-z

**Published:** 2016-06-27

**Authors:** Muzamil Mahdi Abdel Hamid, Arwa F. Elamin, Musab M. Ali Albsheer, Abdelmohaymin A. A. Abdalla, Nouh S. Mahgoub, Shaza O. Mustafa, Mohamed SiddigEltayeb Muneer, Mutaz Amin

**Affiliations:** Department of Parasitology and Medical Entomology, Institute of Endemic Diseases, University of Khartoum, Khartoum, Sudan; Faculty of Medicine, University of Khartoum, Khartoum, Sudan

**Keywords:** *Plasmodium falciparum*, Multiplicity of infection, Genetic diversity, MSP1, MSP2, Severity, Sudan

## Abstract

**Background:**

Multiplicity and genetic diversity of *Plasmodium falciparum* infection might play a role in determining the clinical outcome of malaria infection and could be a fair reflection of the disease transmission rate. This study investigated the genetic diversity of *P. falciparum* and multiplicity of infection in relation to the severity of malaria and age of patients in Gezira State, Sudan.

**Methods:**

A cross-sectional health facilities-based survey was conducted in Gezira State, Sudan in January 2012. A total of 140 *P. falciparum* malaria patients diagnosed with microscopy and confirmed using nested PCR were recruited and classified into uncomplicated malaria and severe malaria states according to the standard WHO criteria. DNA was extracted and MSP1 and MSP2 allelic families were determined using nested PCR.

**Results:**

The overall multiplicity of infection (MOI) was 2.25 and 2.30 and 2.15 for uncomplicated and severe malaria respectively. There were no statistically significant differences between uncomplicated and severe malaria (SM) patient groups in MOI with regard to MSP1, MSP2 and overall MOI (Mann-Whitney U-test; all *P* < 0.05). The predominant MSP1 allelic families were MAD20 for uncomplicated malaria and RO33 for severe malaria. The distribution of both FC27 and IC1/3D7 MSP2 allelic families were approximately the same across disease severity. One hundred and eleven *P. falciparum* isolates (81 %) consisted of multiple genotypes; 71/90 (78.9 %) in uncomplicated malaria and 40/50 (85.1 %) in severe malaria patient groups. Neither MSP1 nor MSP2 allelic families showed association with malaria severity. No statistically significant differences in multiplicity of infection were observed between different age groups.

**Conclusion:**

In this study the majority of *P. falciparum* isolates from uncomplicated and severe malaria patients consisted of multiple genotypes. Further molecular epidemiological studies delineate the link between *P. falciparum* genotype with the malaria phenotype in different regions are encouraged.

## Background

Malaria caused by *Plasmoduim falciparum* is a major public health problem in sub-Saharan countries. In 2015 theglobal burden of the disease reached 214 million cases with 97 countries and 3.2 billion people at risk [1]. In Sudan, malaria is a leading cause of morbidity and mortality. High malaria transmission occurs in 87 % of the population [2]. The peak months of malaria transmission are from September to November. However, in GeziraState there is an additional peak by the end of February, which corresponds to the end of the irrigation season when small pools of water form along the drying canals with subsequent increase in mosquito density [3].

Malaria exhibits a wide range of clinical manifestations ranging from asymptomatic parasitaemia, uncomplicated (mild) to complicated (severe) disease [4]. Molecular epidemiological studies of malaria are used to investigate the genetic diversity of infection with consideration of various factors such as disease phenotype, age and host immunity*. Plasmodium falciparum* is the most virulent malaria parasite species. It exhibits a complex genetic polymorphism which may explain its ability to present with different clinical manifestations of the disease spectrum [5]. Moreover, some have stated that certain *P. falciparum* genotypes can be linked with more virulent infections [6, 7], and the presence of multiple infections may affect the release of different pro-inflammatory cytokines, that makes it more difficult for the immune system to deal with, resulting in severe malaria [8].

Molecular approaches helps to increase our understanding of malaria epidemiology as well as clinical manifestation [9]; these include molecular characterization of the malaria parasite in order to answer why some patients develop severe disease while others end up with mild form. Merozoite Surface proteins 1 and 2 (MSP1 and MSP2) of *P. falciparum* are major blood stage malaria surface antigens [10]. They play a major role in erythrocyte invasion by merozoite parasite [11]; therefore, they are targeted by the immune response [12]. Also they are very suitable markers for identification of genetically distinct parasite sub-populations and investigation of the genetic diversity of *P. falciparum* [13].

MSP1 gene is located on chromosome 9 and contains 17 blocks of sequences, of which 7 are variable, flanked by conserved regions [14]; block 2 is most polymorphic and is grouped into three allelic families MAD20, K1 and RO33 [15]. MSP2 gene is located on chromosome 2 composed of five blocks of which the central block 3 is the most polymorphic [16]. The MSP2 alleles are grouped into two allelic families, FC27 and IC1/3D7) [13].

Several studies have linked MOI to the severity of malaria especially in areas with high transmission rates [17, 18]. In this regard, determination of MOI in a highly endemic area such as the Gezira State becomes crucial, since it predicts clinical course target population with higher attention (e.g. certain age groups). Regular molecular epidemiological surveys that monitor the genetic diversity of *P. falciparum* populations in different regions of the country as well as worldwide and linking parasite genotypes to the disease phenotypes are crucially important.

In this health care service-based study we aimed to investigate the genetic diversity of *P. falciparum* and multiplicity of infection and their relationship to the disease severity and patient age.

## Methods

### Study area

The study was carried out in Wad-MadaniPediatric Hospital (WPH), Wad-Madani teaching hospital (WTH) and neighboring health facilities in Gezira State, located in the center of Sudan. Gezira state lies on the west bank of the Blue Nile. The geographical coordinates of the state are 14°30'0"N, 33°30'0"E. *Plasmodium falciparum* is the predominant species (prevalence 90–95 %), and the *Anopheles arabiensis* is the main mosquito vector [19]. These health facilities were selected because of their high malaria admission rate (they receive malaria patients from all over the state) and good health infrastructure.

### Patients and malaria definition

A total of 140 patients from those screened for malaria during a cross-sectional study conducted in January 2012 were confirmed positive for *P. falciparum* malaria by microscopy and nested PCR. All age groups (from age 1 to 80 years) were targeted. Malaria patients were classified into uncomplicated and severe malaria states according to the WHO criteria [20]. Uncomplicated malaria patients were defined as positive smear for *P. falciparum* and presence of fever (≥ 37.5 °C). Severe malaria patients were having additional symptoms (e.g. convulsions, clinical jaundice, respiratory distress, hyperpasitaemia and severe anemia) [20]. Complicated and severe malaria patients were admitted to the hospital and have been treated. Patients with uncomplicated malaria were recruited from the hospital's general pediatric ward and from the outpatient clinics. Intravenous blood samples (5 ml) were collected in EDTA tubes (vacutainers) at admission before the initiation of anti-malarial treatment. Demographical data were recorded including age and sex.

### Microscopy and parasite counts

Microscopic visualization of both thick and thin blood films was prepared using 10 % Giemsa stain. The stained slides were examined under a light microscope for detection and identification of *Plasmodium* species and for parasite count. Parasite density was determined by counting the number of asexual parasites per 200 white blood cells, and calculated per microliter (μl) using the following formula: number of parasites × 8,000/200, assuming a white blood cell count of 8,000 cells per μl [21].

### Extraction of parasite DNA

Genomic DNA was extracted from 100 μl blood samples or from stored blood spotted on Whatman 3 filter paper using 0.5 % Saponin (Sigma-Aldrich, Taufkirchen, Germany) to free parasites from red blood cells followed by Chelex® 100 (Bio-Rad Laboratories, California, USA) method as described by Plowe et al. [22]. DNA was checked for purity and quantity using Nanodrop spectrophotometer (ND 1,000), and stored at -20 °C until used for PCR amplification.

### Molecular identification, MSP1 and MSP2 genotyping

*Plasmodium* spp. were identified by 18S rRNA gene-based nested PCR using genus- and species-specific primers as described by Snounou et al. [23]. *Plasmodium falciparurm* were further analyzed by two highly polymorphic regions of MSP1 (block 2) and MSP2 (block 3) and their allelic types (MAD20, K1 and RO33) and (FC27and IC1/3D7), respectively, using nested PCR [24]. In brief PCR reactions were carried out in a final volume of 25 μl containing 2 μl of parasite DNA, 2 mM MgCl_2_, 0.2 mMdNTPs, 1 μl of each primer and 1 unit of Taq Polymerase (Vivantis,SelangorDarulEhsan, Malaysia). Cycling conditions for the outer PCR were as follows:initial denaturation at 94 °C for 3 min, followed by 37 cycles of denaturation at 94 °C for 30 s, primer annealing at 55 °C for 1 min; and primer extension at 72 °C for 2 min. The final cycle had a prolonged extension at 72 °C for 10 min. PCR reactions were incubated in a thermal cycler (SensoQuest, Göttingen, Germany). Allelic specific positive control and DNA negative control were included in each set of reactions [25].

### Detection of alleles

The amplified PCR products were analyzed by electrophoresis on 2 % molecular grade agarose gel (Caisson,Utah, USA) and visualized by UV trans-illuminator (BioDoc-It UVP, Cambridge, UK), following ethidium bromide staining. The number and size of DNA fragments was estimated using 100 bp DNA ladder (Vivantis,Selangor Darul Ehsan, Malaysia).

### Multiplicity of infection

Multiplicity of infection (MOI) was determined by calculating the number of different alleles at each locus; single infections were those with only one allele per locus at all of the genotyped loci. Multiclonal infections were defined as those having more than one allele in at least one locus out of the loci genotyped.

### Data analysis

Data were entered and analyzed using the software Statistical Package for Social Sciences version 20 (SPSS, Inc., Chicago, IL, USA). The MSP1 and MSP2 allelic frequency was calculated. The mean multiplicity of infection (MOI) was calculated for MSP1, MSP2 and overall MOI. Continuous variables were compared using Spearman’s rank correlation coefficient. Dichotomous variables were analyzed using Chi-square test. Mann-Whitney U-test was used to compare the mean MOI according to severity (disease phenotype). Different age groups’ MOIs were calculated and the statistical significance was tested using Kruskal-Wallis H-test. Statistical significance level was defined as *P*-value ≤ 0.05 at 95 % confidence interval.

## Results

**Fig. 1:**
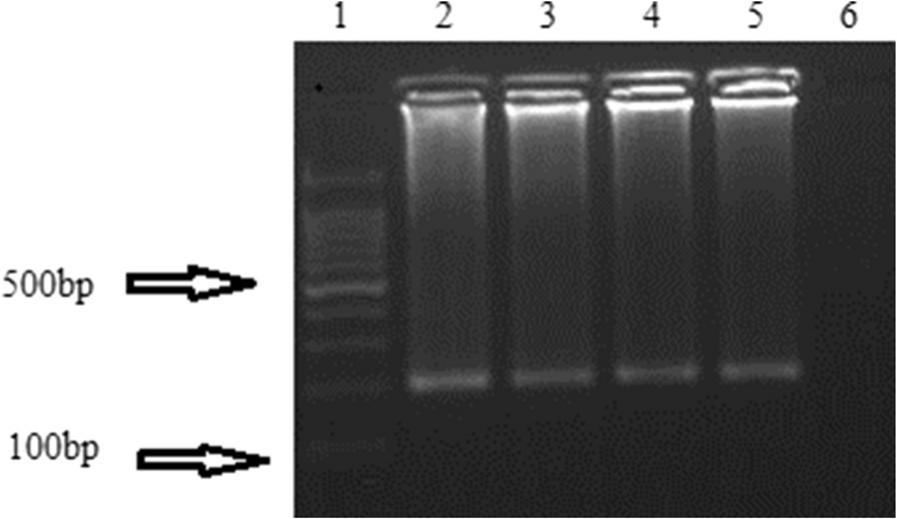
Gel electrophoresis picture for detection of 18S rRNA *P. falciparum* gene: Lane 1, 100 bp DNA ladder; Lanes 2–5, 200 bp18S rRNA gene; Lane 6, negative control

*Plasmodium falciparum* DNA was detected in all malaria patients by nested PCR (Fig. 1). Across all age groups, about two-thirds of malaria patients represented uncomplicated malaria cases and the remaining third were categorized as severe malaria (SM) patients according to the WHO classification. The demographic and clinical data for both uncomplicated and severe malaria patients are summarized in Table 1. All malaria patients were treated using the standard WHO treatment protocol for malaria.

**Table 1:** Demographic and clinical data of uncomplicated and severe malaria patients in Gezira state, Sudan, January 2012

Characteristic	Uncomplicated malaria (*n* = 90)	Severe malaria (*n* = 50)
Age, mean (SD)	23.72 ± 16.69	19.30 ± 14.05
Male gender *n* (%)	55 (76.4)	22 (30.6)
Axillary temperature, mean (SD), °C	37.45 ± 1.29	38.97 ± 1.03
Mean parasite density/μl	5,279.7 (1,956–9,962)	20,940.85 (3,000–74,400)
Hyperpasitaemia (≥50,000/μl of blood)	0 (0)	7 (14)
Respiratory distress (%)	0 (0)	19 (38)
Clinical jaundice *n* (%)	0 (0)	2 (4)
Convulsions	0 (0)	3 (6)
Mean of hemoglobin (g/dl)	12.84 ± 2.16	7.44 ± 2.16
Severe anemia (<5 g/dl) *n* (%)	0 (0)	2 (4)

*Abbreviations*: *n* number of individuals, *SD* standard deviation

### Genotyping of *P. falciparum* and multiplicity of infection in uncomplicated and severe malaria patients

**Fig. 2:**
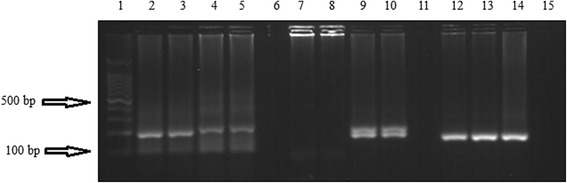
Gel electrophoresis of MSP1 allelic types: Lane 1,100 bp DNA ladder; l. Lanes 2 and 3, 100 bp and 180 bp of MAD20; Lanes 4 and 5, 100 bp and 220 bp of MAD20; Lane 6, negative control of MAD20; Lanes 7 and 8, negative samples of K1; Lanes 9 and 10, 180 bp and 200 bp of K1; Lane 11, negative control of K1; Lanes 12–14, 160 bp of RO33; Lane 15, negative control of RO33

**Fig. 3:**
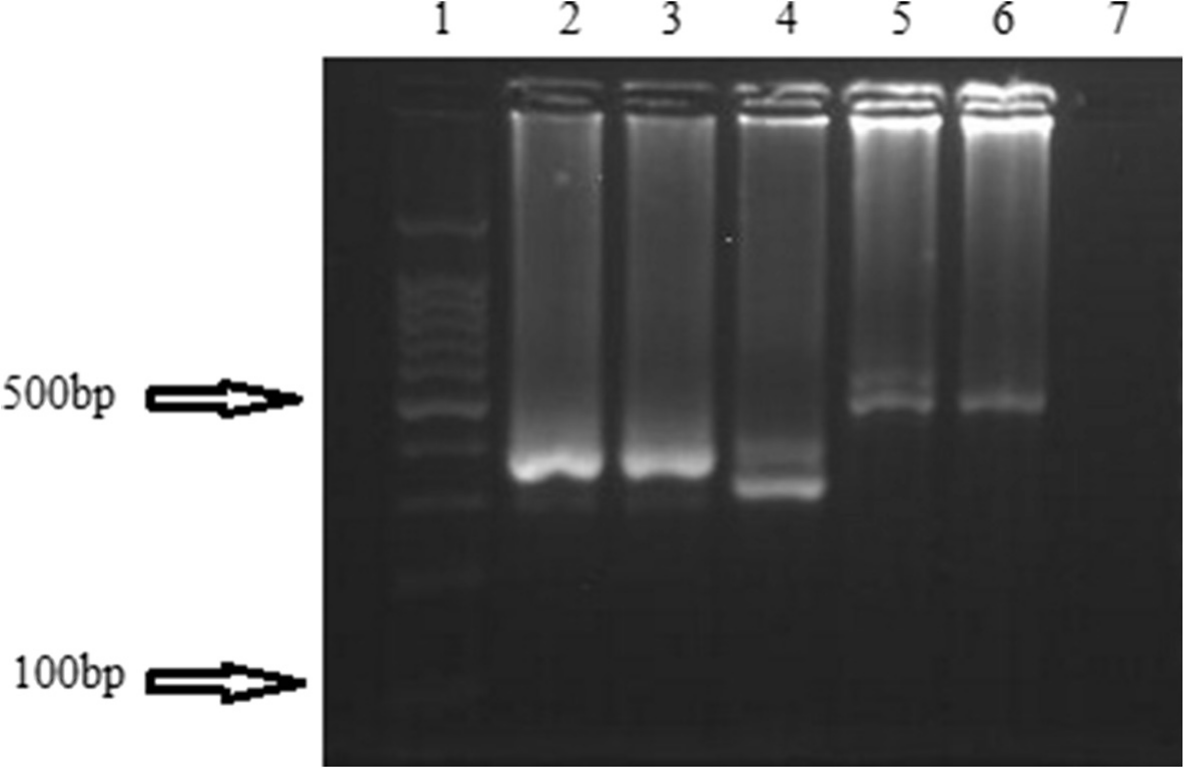
Gel electrophoresis of MSP2 allelic types: Lane 1, 100 bp DNA ladder; Lanes 2–4, 300 bp and 350 bp of FC27; Lane 5, 500 bp and 550 bp and of IC1/3D7; Lane 6, 500 bp of ICI/3D7; Lane 7, negative control

Polymorphism of MSP1 and MSP2 allelic families was assessed in all blood samples. Allelic families were detected in 114/140 (81 %) and 130/140 (92.86 %) samples for MSP1 (Fig. 2) and MSP2 (Fig. 3), respectively.

The overall multiplicity of infection (MOI) was 2.25. The overall MOI with regard to uncomplicated and severe malaria cases were 2.30 (95 % CI: 2.12–2.48) and 2.15 (95 % CI: 1.94–2.36), respectively. However the differences in MSP1, MSP2 and overall MOI were statistically non-significant (Mann-Whitney U-test; *U* =1390.500, 1778.500 and 1934.500, respectively; *P* = 0.826, 0.712 and 0.379, respectively) (Table 2).

**Table 2:** Multiplicity of infection of MSP1 and MSP2 and overall MOI in mild and severe malaria patients in Gezira State, Sudan (January 2012)

Genes	Uncomplicated malaria (*n* = 90)	Severe malaria (*n* = 50)	*P*-value^b^
	MOI (95 % CI)	MOI (95 % CI)	
MSP1	1.95 (1.73–2.16)	1.84 (1.61–2.07)	0.826
MSP2	1.90 (1.72–2.08)	1.83 (1.59–2.08)	0.712
Overall MOI^a^	2.3 (2.12–2.48)	2.15 (1.94–2.36)	0.379

*Abbreviations*: *CI* confidence interval, *MOI* multiplicity of infection

^a^Determined by selecting the highest number of alleles detected in any of the two antigens

^b^ *P*-values from Mann-Whitney U-test

### Allelic frequency and genetic diversity of *P. falciparum* in uncomplicated and severe malaria patients

Allelic genotyping of MSP1 (MAD20, K1 and RO33) and MSP2 (FC27 and ICI/3D7) allelic families showed the polymorphic nature of the Sudanese *P. falciparum* isolates with respect to disease phenotype (uncomplicated *versus* severe malaria patients). In uncomplicated malaria patients, a total of 14 different-sized alleles for MSP1 were detected compared to 21 different-sized alleles for MSP2 (Table 3).

**Table 3:** MSP1, MSP2 genetic diversity, allelic frequency and allelic families fragment sizein mild and severe malaria patients in Gezira State, Sudan (January 2012)

	Uncomplicated malaria (*n* = 90)			Severe malaria (*n* = 50)			
Gene/Allele	Positive *n* (%)	Fragment size (bp)	No. of alleles	Positive *n* (%)	Fragment size (bp)	No. of alleles	*P*-value*
MSP1							
MAD20	16 (17.8)	100–380	7	3 (6)	100–300	7	
K1	5 (5.6)	160–300	6	5 (10)	180–300	4	
RO33	13 (14.4)	160	1	3 (6)	160	1	
MAD20/K1	10 (11.1)			4 (8)			
MAD20/RO33	14 (15.5)			8 (16)			
K1/RO33	9 (10.0)			8 (16)			
MAD20/K1/RO33	10 (11.1)			6 (12)			
Total	77 (85.5)		14	37 (74)		12	0.326
Total MAD20	50 (55.5)			21 (42)			0.078
Total K1	34 (38.0)			23 (46)			0.343
Total RO33	46 (51.0)			25 (50)			0.724
MSP2							
FC27	20 (22.2)	200–600	10	11 (22)	200–600	9	
IC1/3D7	19 (21.1)	250–700	11	8 (16)	350–700	11	
FC27/IC1/3D7	49 (54.4)			23 (46)			
Total	88 (97.7)		21	42 (84)		20	0.002
Total FC27	69 (77.0)			34 (68)			0.265
Total IC1/3D7	68 (76.0)			31 (62)			0.091

*P-values using Chi-square test

*Abbreviation*: *n* number of individuals

In severe malaria patients, a total number of 12 different-sized alleles for MSP1 were detected compared to 20 different-sized alleles for MSP2. The predominant MSP1 allelic families were MAD20 for the uncomplicated malaria and RO33 for the severe malaria. The distribution of both FC27 and IC1/3D7 MSP2 allelic families was approximately the same across disease severity. One hundred and eleven *P. falciparum* isolates (81 %) consisted of multiple genotypes; 71/90 (78.9 %) in uncomplicated malaria and 40/50 (85.1 %) in severe malaria patients. Neither MSP1 nor MSP2 allelic families showed association with malaria severity. The frequency of the identified MSP1 and MSP2 allelic families across the uncomplicated and severe malaria patients is shown in Table 3.

### Multiplicity of infection, malaria phenotype, clonality of isolates, parasite density and different distribution of allelic families across different age groups

Approximately more than three-quarters of malaria patients in all age groups were found to have a multiclonal *P.falciparum* infections (Table 4). Multiplicity of infection was the highest (2.6, 95 % CI: 2.02–3.18) in the youngest age group (< 5 year-old) (Table 4): however the result wasnot statistically significant (Spearman’s rank coefficient = 0.04; *P* = 0.658). There was a statistically significant positive correlation between age and parasite density (Spearman’s rank coefficient = 0.263; *P* = 0.046). The oldest age group (> 40 year-old) had the highest mean parasite density (24,357.8 parasites/μl) (Table 4). The distribution of different MSP1 and MSP2 allelic families and their different combinations are shown in Table 4. The difference in distribution was only significant for total MSP2 (Chi-square Test, *Χ*^2^ = 8.673, *P* = 0.034).

**Table 4:** Distribution of *Plasmodium falciparum* MSP1 and MSP2 allelic families and mean of parasite density among age groups of uncomplicated and severe *P. falciparum* malaria patients in Gezira State, Sudan (January 2012)

Gene/Allele	Age group (years)				
	<5	5–19	20–40	>40	Total
	*n* (%)	*n* (%)	*n* (%)	*n* (%)	*n* (%)
	(*n* = 16)	(*n* = 53)	(*n* = 52)	(*n* = 19)	(*n* = 140)
MSP1					
MAD20	0 (0)	10 (18.9)	6 (11.5)	3 (15.8)	19 (16.7)
K1	1 (6.3)	4 (7.5)	4 (7.7)	1 (5.3)	10 (8.8)
RO33	1 (6.3)	4 (7.5)	11 (21.2)	0 (0)	16 (14.0)
MAD20/K1	3 (18.7)	3 (5.7)	5 (9.6)	3 (15.8)	14 (12.3)
MAD20/RO33	2 (12.5)	10 (18.9)	6 (11.5)	4 (21.0)	22 (19.3)
K1/RO33	0 (0)	4 (7.5)	9 (17.3)	4 (21.0)	17 (14.9)
MAD20/K1/RO33	4 (25.0)	7 (13.2)	3 (5.8)	2 (10.5)	16 (14.0)
Total	11 (68.8)	42 (79.2)	44 (84.6)	17 (89.4)	114 (100)
MSP2					
FC27	2 (12.5)	13 (24.5)	11 (21.2)	5 (26.3)	31 (23.9)
IC1/3D7	3 (18.8)	10 (18.9)	9 (17.3)	5 (26.3)	27 (20.8)
FC27/IC1/3D7	8 (50.0)	24 (45.3)	31 (59.6)	9 (47.4)	72 (55.4)
Total	13 (10.0)	47 (36.2)	51 (39.2)	19 (14.6)	130 (100)
Multiclonal isolates	14 (87.5)	40 (80.0)	41 (78.9)	16 (84.2)	111 (79.3)
Mild malaria *n* (%)	10 (60.0)	32 (60.0)	35 (67.0)	13 (68.0)	90 (64.3)
Severe malaria *n* (%)	6 (40.0)	21 (39.0)	17 (32.0)	6 (31.0)	50 (35.7)
Overall MOI	2.6	2.1	2.2	2.4	
Parasite density (no. of parasites/μl)	5,859.7	14,630.8	13,251.4	24,357.8	

*Abbreviations*: *n* number of individuals, *MOI* multiplicity of infection

## Discussion

In Sudan, very few studies investigated the genetic diversity and multiplicity of infection of *P. falciaprum*. To our knowledge, this is the first study to provide data on the genetic diversity and multiplicity of *P. falciparum* isolates in relation to disease phenotype based on MSP antigen in Gezira State, Central Sudan. In our study there was no association between MOI and malaria outcome (severe *versus* uncomplicated) which is consistent with other reports from Sudan [26], Ghana [27], Gabon [28], Gambia [29] and Madagascar [30], but our results differ from those reporting higher MOI in severe malaria patients in Uganda [17] and India [18] and those reporting higher MOI in uncomplicated malaria patients in Senegal [31] and Nigeria [32]. However the methodology used in our study (due to our limited funds) is comparatively weak because variation in size at MSP loci may be hard to interpret since multiple insertion-deletion mutations, recombination events, and/or convergence due to selection could generate alleles that differ at the sequence level but have the same fragment size or restriction fragment length polymorphism pattern and the results should be confirmed by DNA sequencing.

Our reported overall MOI (2.25) was higher than that observed in Gadarif state, Sudan(overall MOI = 1.52) [26] and lower than recorded in a study conducted in Mauritania (overall MOI = 3.2) where malaria is hyperendemic [33]. This conflict of results is most likely due to inter-laboratory variation which restricts comparison of MOI in different settings [34].

In this study we found a remarkably high frequency of multiclonal isolates (81 %) comparable to that (82.3 %) reported in Mauritania [33]. The frequency of multiclonal isolates was higher in severe compared to uncomplicated malaria patients (85.1 *vs* 78.9 %). This observation is in agreement with the study of Shigidi et al. [35] which reported 58.6 % and 35.6 % in severe and uncomplicated malaria patients, respectively, and in contrast to the results of A-Elbasit et al. [26], who observerd 34 % and 41 % in severe and uncomplicated malaria patients, respectively. These differences in multiclonal infections proportions could be attributed to different geographical regions. It is interesting to note that the current study was conducted after implementation of artemisinin combination therapy (ACT) in Sudan [36]. In this study we found no association between *P. falciparum* specific allele families and clinical outcome and several studies reached the same conclusion [17, 18, 26, 28, 30].

The observed increase in multiplicity of infection and frequency of multiclonal isolates of *P. falciparum* in this study area compared to previous studies in Sudan could be due to some of the following reasons: (i) the followed treatment guidelines in Sudan using ACTas a treatment regimen since 2004 [36] which doesnot affect parasite mature sexual stages in which the genetic re-assortments and re-arrangements occur; (ii) missed treated malaria patients;(iii) non-adherence to antimalarial drugs and/or eographical differences.

With regard to MSP1, inuncomplicated malaria patients the predominant allelic family was MAD20, whearas in severe malaria patients the predominant allelic family was RO33. These results are in contrast to what was reported by Bouyou-Akotet et al. in Gabon [28]. On the other hand the distribution of MSP2 for both FC27 and IC1/3D7 was approximately the same across the uncomplicated and severe malaria patients which agrees with A-Elbasit et al. [26] who obtained similar results in Sudan. These differences are interpreted cautiously as study areas are different which poses different malarial endemicity.

The current study found no association between multiplicity of infection and patients’ age group. This is in agreement with previous studies conducted in Muritania [33], Senegal [31] and Benin [37]. However, this finding contrasts with reports from other locations in Burkina Faso [38], Senegal [31] and Tanazania [39]. Previous studies on the varaiation of multiplicity of infection with regard to agedistribution highlighted that the effect of age on the multiplicity of infection is considerably affected by the endemicity of malaria which is most likely a reflection of the development of antiparasite specific immunity [40].

## Conclusions

In this study the multiplicity of infection was comparable between uncomplicated and severe malaria patients. The multiplicity of infection and the distribution of multiclonal isolates of *P. falciparum* were higher than previous studies in Sudan. Neither MSP1 nor MSP2 allelic families exhibited significant association with malaria severity. There was no statistically significant difference between different age groups with regard to the multiplicity of infection. The observed high multiclonal isolates may facilitate the spread of resistant strains with time. Further molecular epidemiological studies that would help delineate the link between *P. falciparum* genotypes with the malaria phenotypes in different regions are encouraged.

## Abbreviations

MOI, multiplicity of Infection; MSP1, merozoite surface protein 1; MSP2, Merozoite surface protein 2; PCR, polymerase chain reaction

## References

[1] World Health Organization. World Malaria Report (2008–2015). Geneva. World Health Organization; 2015. Available in [http://www.who.int/malaria/publications/world_malaria_report/en/].

[2] World Health Organization. World Malaria Report (2008–2015). Regional and country profiles. Geneva. World Health Organization; 2015. Available in [http://www.who.int/malaria/publications/world-malaria-report-2015/wmr2015 profiles.pdf?ua = 1]

[3] Petrarca V, Nugud AD, Ahmed MA, Haridi AM, Di Deco MA, Coluzzi M (2000). Cytogenetics of the *Anopheles gambiae* complex in Sudan, with special reference to *An. arabiensis*: relationships with East and West African populations. Med Vet Entomol.

[4] Miller LH, Baruch DI, Marsh K, Doumbo OK (2002). The pathogenic basis of malaria. Nature.

[5] Mendis KN, Carter R (1995). Clinical disease and pathogenesis in malaria. Parasitol Today.

[6] Engelbrecht F, Felger I, Genton B, Alpers M, Beck HP (1995). *Plasmodium falciparum* malaria morbidity is associated with specific merozoite surface antigen 2 genotypes. Exp Parasitol.

[7] Ariey F, Hommel D, Le Scanf C, Duchemin JB, Peneau C, Hulin A (2001). Association of severe malaria with a specific *Plasmodium falciparum* genotype in French Guiana. J Infect Dis.

[8] John CC, Park GS, Sam-Agudu N, Opoka RO, Boivin MJ (2008). Elevated serum levels of IL-1ra in children with *Plasmodium falciparum* malaria are associated with increased severity of disease. Cytokine.

[9] Brown GV, Beck HP, Molyneux M, Marsh K (2000). Molecular approaches to epidemiology and clinical aspects of malaria. Parasitol Today.

[10] Chitarra V, Holm I, Bentley GA, Petres S, Longacre S (1999). The crystal structure of C-terminal merozoite surface protein 1 at 1.8 resolution, a highly protective malaria vaccine candidate. Mol Cell.

[11] Holder AA, Blackman MJ, Burghaus PA, Chappel JA, Ling IT, McCallum-Deighton N (1992). A malaria merozoite surface protein (MSP1)-structure, processing and function. Mem Inst Oswaldo Cruz.

[12] Apio B, Nalunkuma A, Okello D, Riley E, Egwang TG (2000). Human IgG subclass antibodies to the 19 kilo daltoncarboxy terminal fragment of *Plasmodium falciparum* merozoite surface protein 1 (MSP-119) and predominance of the MAD20 allelic type of MSP-1 in Uganda. East Afr Med J.

[13] Mohammed H, Mindaye T, Belayneh M, Kassa M, Assefa A, Tadesse M (2015). Genetic diversity of *Plasmodium falciparum* isolates based on MSP-1 and MSP-2 genes from Kolla-Shele area, ArbaminchZuria District, southwest Ethiopia. Malar J.

[14] Takala S, Branch O, Escalante AA, Kariuki S, Wootton J, Lal AA (2002). Evidence for intragenic recombination in *Plasmodium falciparum*: identification of a novel allele family in block 2 of merozoite surface protein-1: Asembo Bay Area Cohort Project XIV. MolBiochemParasitol.

[15] Takala SL, Escalante AA, Branch OH, Kariuki S, Biswas S, Chaiyaroj SC (2006). Genetic diversity in the Block 2 region of the merozoite surface protein 1 (MSP-1) of *Plasmodium falciparum*: additional complexity and selection and convergence in fragment size polymorphism. Infect Genet Evol.

[16] Ferreira MU, Hartl DL (2007). *Plasmodium falciparum*: worldwide sequence diversity and evolution of the malaria vaccine candidate merozoite surface protein-2 (MSP-2). ExpParasitol.

[17] Kiwuwa MS, Ribacke U, Moll K, Byarugaba J, Lundblom K, Farnert A (2013). Genetic diversity of *Plasmodium falciparum* infections in mild and severe malaria of children from Kampala. Uganda Parasitol Res.

[18] Rout R, Mohapatra BN, Kar SK, Ranjit M (2009). Genetic complexity and transmissibility of *Plasmodium falciparum* parasites causing severe malaria in central-east coast India. Trop Biomed.

[19] Ranson H, Abdallah H, Badolo A, Guelbeogo WM, Kerah-Hinzoumbé C, Yangalbé-Kalnoné E (2009). Insecticide resistance in *Anopheles gambiae*: data from the first year of a multi-country study highlight the extent of the problem. Malar J.

[20] World Health Organization (2000). Severe *falciparum* malaria. Trans R Soc Trop Med Hyg.

[21] Gilles HM. The differential diagnosis of malaria. In: Wernsdorfer WH and McGregor IA, editors. Malaria: Principles and Practice of Malariology. Edinburgh: Churchill Livingstone; 1988;1:769–79.

[22] Plowe CV, Djimde A, Bouare M, Doumbo O, Wellems TE (1995). Pyrimethamine and proguanil resistance-conferring mutations in *Plasmodium falciparum* dihydrofolatereductase: polymerase chain reaction method for surveillance in Africa. Am J Trop Med Hyg.

[23] Snounou G, Viriyakosol S, Jarra W, Thaithong S, Brown KN (1993). Identification of the four human malaria parasite species in field samples by the polymerase chain reaction and detection of a high prevalence of mixed infections. MolBiochemParasitol.

[24] Ntoumi F, Ngoundou-Landji J, Lekoulou F, Luty A, Deloron P, Ringwald P (2000). Site-based study on polymorphism of *Plasmodium falciparum* MSP1 and MSP2 genes in isolates from two villages in Central Africa. Parassitologia.

[25] Hamid MM, Mohammed SB, El Hassan IM (2013). Genetic diversity of *Plasmodium falciparum* field isolates in central Sudan inferred by PCR genotyping of Merozoite surface protein 1 and 2. N Am J Med Sci.

[26] A-Elbasit IE, ElGhazali G, A-Elgadir TM, Hamad AA, Babiker HA, Elbashir MI, Giha HA (2007). Allelic polymorphism of MSP2 gene in severe *P. falciparum* malaria in an area of low and seasonal transmission. Parasitol Res.

[27] Nielsen MA, Staalsoe T, Kurtzhals JA, Goka BQ, Dodoo D, Alifrangis M (2002). *Plasmodium falciparum* variant surface antigen expression varies between isolates causing severe and non-severe malaria and is modified by acquired immunity. J Immunol.

[28] Bouyou-Akotet MK, M’Bondoukwé NP, Mawili-Mboumba DP (2015). Genetic polymorphism of merozoite surface protein-1 in *Plasmodium falciparum* isolates from patients with mild to severe malaria in Libreville. Gabon Parasite.

[29] Conway DJ, Greenwood BM, McBride JS (1991). The epidemiology of multiple-clone *Plasmodium falciparum* infections in Gambian patients. Parasitology.

[30] Durand R, Ariey F, Cojean S, Fontanet A, Ranaivo L, Ranarivelo LA (2008). Analysis of circulating populations of *Plasmodium falciparum* in mild and severe malaria in two different epidemiological patterns in Madagascar. Trop Med Int Health.

[31] Konaté L, Zwetyenga J, Rogier C, Bischoff E, Fontenille D, Tall A (1999). Variation of *Plasmodium falciparum* msp1 block 2 and msp2 allele prevalence and of infection complexity in two neighbouring Senegalese villages with different transmission conditions. Trans R Soc Trop Med Hyg.

[32] Amodu OK, Oyedeji SI, Ntoumi F, Orimadegun AE, Gbadegesin RA, Olumese PE, Omotade OO (2008). Complexity of the msp2 locus and the severity of childhood malaria, in south-western Nigeria. Ann Trop Med Parasitol.

[33] Ahmedou MS, Ndiaye M, Abdallahi MO, Lekweiry KM, Bogreau H, Konaté L (2014). Polymorphism of the merozoite surface protein-1 block 2 region in *Plasmodium falciparum* isolates from Mauritania. Malaria J.

[34] Färnert A, Arez AP, Babiker HA, Beck HP, Benito A, Björkman A (2001). Genotyping of *Plasmodium falciparum* infections by PCR: a comparative multicentre study. Trans R Soc Trop Med Hyg.

[35] Shigidi MM, Hashim RA, Idris MN, Mukhtar MM, Sokrab TO (2004). Parasite diversity in adult patients with cerebral malaria: A hospital-based, case–control study. Am J Trop Med Hyg.

[36] Malik EM, Mohamed TA, Elmardi KA, Mowien MM, Elhassan AH, Elamin SB (2006). From chloroquine to artemisinin based combination therapy: The Sudanese experience. Malar J.

[37] Issifou S, Djikou S, Sanni A, Lekoulou F, Ntoumi F (2001). Pas d’influence de la saison de transmission ni de l´âge des patients sur la complexité et la diversitégénétique des infections dues à *Plasmodium falciparum* à Cotonou (Bénin). Bull SocPatholExot.

[38] Soulama I, Nébié I, Ouédraogo A, Gansane A, Diarra A, Tiono AB (2009). *Plasmodium falciparum* genotypes diversity in symptomatic malaria of children living in an urban and rural setting in Burkina Faso. Malar J.

[39] Smith T, Beck HP, Kitua A, Mwankusye S, Felgei I, Fraser-Hurt N (1999). Age dependence of the multiplicity of *Plasmodium falciparum* infections and of other malariological indices in an area of high endemicity. Trans R Soc Trop Med Hyg.

[40] Vafa M, Troye-Blomberg M, Anchang J, Garcia A, Migot-Nabias F (2008). Multiplicity of *Plasmodium falciparum* infection in asymptomatic children in Senegal: relation to transmission, age and erythrocyte variants. Malar J.

